# Structural and Biochemical Characterization of the *Francisella tularensis* Pathogenicity Regulator, Macrophage Locus Protein A (MglA)

**DOI:** 10.1371/journal.pone.0128225

**Published:** 2015-06-29

**Authors:** Bonnie J. Cuthbert, Richard G. Brennan, Maria A. Schumacher

**Affiliations:** Department of Biochemistry, Duke University School of Medicine, Durham, North Carolina, 27710, United States of America; University of Louisville, UNITED STATES

## Abstract

*Francisella tularensis* is one of the most infectious bacteria known and is the etiologic agent of tularemia. Francisella virulence arises from a 33 kilobase (Kb) pathogenicity island (FPI) that is regulated by the macrophage locus protein A (MglA) and the stringent starvation protein A (SspA). These proteins interact with both RNA polymerase (RNAP) and the pathogenicity island gene regulator (PigR) to activate FPI transcription. However, the molecular mechanisms involved are not well understood. Indeed, while most bacterial SspA proteins function as homodimers to activate transcription, *F*. *tularensis* SspA forms a heterodimer with the MglA protein, which is unique to *F*. *tularensis*. To gain insight into MglA function, we performed structural and biochemical studies. The MglA structure revealed that it contains a fold similar to the SspA protein family. Unexpectedly, MglA also formed a homodimer in the crystal. Chemical crosslinking and size exclusion chromatography (SEC) studies showed that MglA is able to self-associate in solution to form a dimer but that it preferentially heterodimerizes with SspA. Finally, the MglA structure revealed malate, which was used in crystallization, bound in an open pocket formed by the dimer, suggesting the possibility that this cleft could function in small molecule ligand binding. The location of this binding region relative to recently mapped PigR and RNAP interacting sites suggest possible roles for small molecule binding in MglA and SspA•MglA function.

## Introduction

The Gram-negative intracellular bacterium *Francisella tularensis* is the causative agent of tularemia and is one of the most virulent pathogens known. Due to its potential to be deployed as a bioterroist agent, *F*. *tularensis* has been classified as a category A bioweapon by the United States government. The virulence of *F*. *tularensis* stems largely from a 33 Kb pathogenicity island, abbreviated as FPI, on the bacterial genome. The FPI contains 16 to 19 open reading frames [1,2]. These genes are thought to encode a type VI-like protein secretion system that mediates intra-macrophage growth [3,4]. Indeed, disruption of FPI transcription results in avirulent *F*. *tularensis* and poor intracellular survival of the pathogen. Regulation of the FPI has been shown to require the stringent starvation protein A (SspA) and the macrophage growth locus protein A (MglA) [5]. Data showing that SspA and MglA form a heterodimer that binds RNA Polymerase (RNAP) indicate that these proteins play a central role in *F*. *tularensis* virulence and suggest that the SspA•MglA complex functions in recruitment and/or assembly of an active RNAP. Interestingly, SspA•MglA activity has also been linked to the stringent response, which results from amino acid starvation and leads to the production of the alarmone, guanosine-tetraphosphate and guanosine-pentaphosphate, (p)ppGpp [6]. How the stringent response ties into SspA•MglA-mediated transcription activation functions remains unclear, however involvement of the transcription regulator called the pathogenicity island gene regulator (PigR, also known as FevR) has been suggested [6–11].

Several studies have shown that SspA•MglA binds not only to PigR, but also to the transcription regulator PmrA [6–11]. Notably, PigR and PmrA belong to distinct structural families; PigR is proposed to be a MerR family member while PmrA is a response regulator, the activity of which is effected by phosphorylation. As PigR and PmrA are putative DNA binding proteins that interact with SspA•MglA, it has been postulated that they could act as bridges to recruit RNAP directly to the FPI [6,12,13]. However, a recent study employing ChIP-Seq analyses revealed the remarkable finding that SspA•MglA, PigR and RNAP appear to be docked at all *F*. *tularensis* promoters [12]. It seems unlikely that PigR interactions with DNA mediate the specific tethering of SspA•MglA to all these promoters. Indeed, these studies also showed that specific DNA sequences, called PRE (PigR Response Element), were found only in promoters that were activated by SspA•MglA and PigR, indicating that PigR may directly bind this sequence to activate transcription, in some unknown manner, from select virulence promoters.

In addition to *F*. *tularensis* [14], SspA proteins have been shown to be associated with the virulence of several bacteria includin*g Neisseria gonorrhoeae* [15], *Vibrio cholerae* [16,17] and enterohaemorrhagic *Escherichia coli* (*EHEC*) [18]. The *E*. *coli* SspA protein is the best-characterized member of this family and was shown to be required for acid resistance and transcriptional activation of phage P1 late genes [19,20]. *E*. *coli* SspA appears to function by interacting with and recruiting *E*. *coli* RNAP to specific promoter sites [5,21,22]. Interestingly, a recent report suggested that *Pseudomonas aeruginosa* SspA could function as an anti-sigma factor [23]. Thus, these combined data suggest that SspA proteins mediate their transcriptional effects by directly binding RNAP to regulate its function.

Structures have been determined for the SspA proteins from *Haemophilus influenza*, *Pseudomonas fluorescens*, *Pseudomonas putida* and *Yersinia pestis* and reveal that SspA is a member of the cytosolic glutathione transferase (cGST) superfamily. GST proteins have been categorized into numerous classes based on structural homologies and are found in a range of organisms, from bacteria to humans [24]. These proteins catalyze the nucleophilic addition of the sulfur atom from reduced glutathione (GSH) to the electrophilic groups of hydrophobic molecules. The vast majority of GST proteins are homodimers, however heterodimeric GSTs have been reported [24]. GST proteins are composed of two domains: a thioredoxin-like N-terminal domain and a larger C-terminal domain. The N-terminal domain includes the majority of the GSH binding site, while the C-terminal domain contains a binding pocket for hydrophobic co-substrates.

Although SspA proteins are structurally similar to GST proteins they lack GST activity [25]. SspA proteins also differ from canonical GST proteins in their dimerization modes. SspA proteins harbor less extensive dimer interfaces, whereby one side of the molecule mediates most of the dimer contacts, leaving the other side splayed open. The compact “top” of the dimer contains residues implicated in RNAP binding [25]. *F*. *tularensis* SspA is similar to other SspA proteins in that it is involved in regulating transcription by interacting with RNAP. However, in contrast to other SspAs, the *F*. *tularensis* protein forms heterodimers with MglA to effect transcription [5]. To gain insight into the structure and function of an MglA protein, which is unique to *Francisella*, we carried out crystallographic and biochemical studies.

## Materials and Methods

### Expression and purification of MglA

A gene encoding the *F*. *tularensis* ssp. *holarctica* MglA that was codon optimized for expression in *E*. *coli* was purchased from Genscript (Piscataway, NJ). This gene was cloned into the pMCSG9 vector such that it expressed a cleavable MBP tag fused to its N-terminus [26]. The MglA-MBP expressing plasmid was transformed into C41(DE3) cells. For protein expression, the cells were grown at 37°C to an OD_600_ of 0.6–0.8 and induced with 0.5 mM isopropyl β-D-1-thio-galactopyranoside (IPTG). After IPTG addition, the temperature was reduced to 15°C and the cells grown overnight. The harvested cells were stored at -80°C until use. Cells were lysed in 20 mM Tris, pH 7.5, 200 mM NaCl, 10% glycerol, and 7.5 mM imidazole, 1 mM β-mercaptoethanol, 2 mg/L DNase I, and 1 mM PMSF using a microfluidizer. Cell debris was removed by centrifugation at 34,960 xg. The clarified supernatant was loaded onto a Ni-NTA column and washed with increasing concentrations of imidazole in Buffer A (20 mM Tris, pH 7.5, 200 mM NaCl, 10% glycerol, and 1 mM β-mercaptoethanol). The protein was eluted using Buffer A containing 0.25 to 2.0 M imidazole. To remove the MBP-tag, the protein was first buffer exchanged into Buffer A and then subjected to His_6_-TEV digestion overnight at room temperature. The treated protein sample was applied to a Ni-NTA column, which removed the tag, His_6_-TEV and uncleaved fusion protein. MglA was collected in the flow through and the protein further purified via size exclusion chromatography (SEC) using a Superdex S75 column. The buffer used for SEC was 20 mM Tris, pH 7.5, 200 mM NaCl, 10% glycerol, and 1 mM dithiothreitol (DTT). The protein purity was assessed from SDS-PAGE analysis and the protein concentration was determined using Bradford assays.

Selenomethionine-substituted MglA protein was produced for phase determination by the methionine inhibitory pathway. Briefly, an overnight culture grown in Luria Broth (LB) and expressing MglA was centrifuged and the cell pellet thoroughly washed with M9 media to remove any methionine. Washed cells were then resuspended in M9 media and used to inoculate 1.5 L flasks of M9 media. The cells were grown to an OD_600_ of ~0.4 at which point 50 mg/L selenomethionine and 40 mg/L amino acids that inhibit selenomethionine biosynthesis were added and the temperature dropped to 15°C. The cells were grown for an additional 15 min and 0.5 mM IPTG was then added to induce production of selenomethionine-substituted MglA. The cells were grown overnight and collected the next day by centrifugation. The purification of selenomethionine-substituted MglA was performed as for the wild type protein.

### Expression and purification of SspA•MglA and a GST control protein

The *F*. *tularensis* SspA expression plasmid was provided by the Lorca lab (University of Florida) [27]. For co-expression of MglA and SspA, the MglA-MBP construct was dicistronically cloned with *sspA* into pET28a and transformed into C41(DE3). In order to achieve this, both genes were amplified using PCR with primers encoding an *Nco*I site at the 5' end, and tandem *Nhe*I and *Bam*HI sites at the 3' end. Both genes were ligated into pET28a using *Nco*I and *Bam*HI, *sspA* was then excised from pET28a using *Xba*I and *Bam*HI and ligated into the *mglA-MBP* bearing pET28a. The resulting vector encodes both MglA-MBP and untagged SspA with an intergenic linker containing a ribosome-binding site allowing for expression of both genes. For protein expression, 1.5 L cultures of the cells were grown at 37°C until an OD_600_ of 0.6–0.8 was reached. Protein expression was then induced by addition of 0.5 mM IPTG. Cells were harvested 3 hrs after induction and stored at -80°C. SspA•MglA was purified by the same protocol used to purify MglA. Both proteins are present at equal concentrations as shown by SDS-PAGE analysis. The glutathione-S-transferase (GST) expressed from pET41a was used as the control in transferase activity assays. The protein was expressed and purified using the same protocol used to purify SspA•MglA, with the exception that no TEV digestion was necessary.

### GST activity assays

The glutathione transferase activity of MglA, SspA•MglA and GST was determined by a continuous spectrophotometric assay, which follows the conjugation of 1-chloro-2,4-dinitrobenzene (CDNB) to reduced GSH. Reactions were performed at 25°C in 1.5 mL reaction volumes, with concentrations of 97 mM potassium phosphate, 0.97 mM ethylenediaminetetraacetic acid (EDTA), 2.5 mM reduced GSH, 1 mM CDNB, and 3.2% ethanol. For each protein, the above reagents were mixed and after the baseline was established, 5 μg of protein (either GST, MglA or SspA•MglA) was added to the sample. The resulting increase in absorption at 340 nm was measured for 5 min. As a control, a blank reaction was prepared without the addition of enzyme and the absorption at 340 nm was recorded. Enzyme activities were calculated using the equation:

 Enzyme (units/mL)=1.5 mL reaction ×( Test(A340min) - Blank(A340min) )9.6×0.05 mL of enzyme used

A_340_ / min was obtained using the maximum linear rate for both the test and the blank sample. In the reaction, 1.5 mL represents the total reaction volume, and 9.6 is the millimolar extinction coefficient of CDNB at 340 nm. One unit will conjugate 1 μmole of CDNB per minute at pH 6.5 at 25°C per mg of protein.

### Size exclusion chromatography (SEC) studies

For SEC studies on MglA, MglA-MBP and SspA•MglA, proteins at ~3 mg/mL were injected onto a Superdex S75 column pre-equilibrated with 20 mM Tris pH 7.5, 250 mM NaCl, 10% glycerol and 1 mM DTT. Molecular weights for the peaks were extrapolated using the resultant elution volumes for protein standards run on the Superdex S75 column.

### Protein crosslinking-SEC experiments

For SEC analysis of glutaraldehyde-crosslinked MglA, 20 mg of purified MglA at 0.5 mg/mL in 50 mM Hepes, pH 7.5, 50 mM NaCl, and 0.1 mM EDTA were crosslinked with 4 mL of 2.3% glutaraldehyde for 5 min. The reaction was quenched with 4 mL of 2.0 M Tris, pH 7.5. The crosslinked protein was concentrated to 5 mg/mL before injection onto the Superdex S75 column. The buffer used for SEC was 20 mM Tris, pH 7.5, 250 mM NaCl. The triple MglA mutant, MglA(D59R-E70R-D74R) was made by QuikChange following the protocol provided by the manufacturer and purified as per the wild type protein. The purified mutant protein was subjected to crosslinking and SEC following the same methods used for the wild type protein. The majority of the mutant MglA protein (>70%) was lost during crosslinking to precipitation. SEC was performed with 1.4 mg of DED MglA. The sample elution volumes of the proteins were plotted against a standard curve to determine the relative molecular weights of the samples.

### Structure determination of MglA

Crystals of MglA and selenomethionine-substituted MglA were grown using 2.1 M Malic acid, pH 7.0 as the crystallization solution. The structure was determined using multiple wavelength anomalous diffraction (MAD) from data collected on a selenomethionine-substituted MglA crystal. For the MAD experiment, X-ray intensity data were collected at three wavelengths (peak, inflection and remote) at ALS beamline 8.3.1 (Table 1). The images were integrated using Mosflm [28] and scaled with SCALA [29,30]. The heavy atom substructure was obtained using SOLVE and phases and density modification carried out in CNS. Although the map was of excellent quality, the resolution was not sufficient to permit autotracing [31,32]. Hence, the structure was constructed manually using Coot [33]. The structure was refined with phenix.refine to final R_work_ and R_free_ values of 17.3% and 22.8%, respectively [34]. The final structure includes residues 1–201 and 1–197 for two MglA molecules in the crystallographic asymmetric unit (ASU), 2 malate molecules and 70 water molecules. [35]. Final refinement statistics are listed in Table 1.

**Table 1:** Summary of MglA data collection, processing and refinement statistics.

	MglA Selenomethionine		
	Peak	Remote	Inflection
Data collected at	ALS	ALS	ALS
Wavelength (eV)	12657	12957	12655
Resolution range (Å)	100.04–2.95	100.05–2.88	100.05–2.98
	(3.11–2.95)^a^	(3.04–2.88)	(3.14–2.98)
Space group	P6_1_	P6_1_	P6_1_
a, b, c (Å)	104.7, 104.7, 100.5	104.7, 104.7, 100.5	104.7, 104.7, 100.5
α, β, γ (°)	90, 90, 120	90, 90, 120	90, 90, 120
Completeness (%)	99.8 (100)	99.8 (100)	99.8 (100)
Redundancy	3.6 (3.6)	3.6 (3.7)	3.6 (3.7)
I/σI	11.2 (3.2)	10.9 (2.6)	11.7 (3.6)
R_sym_ ^b^	0.07 (0.35)	0.07 (0.43)	0.06 (0.31)
**Refinement and protein geometry analysis** ^**c**^			
R_work_ ^d^/R_free_ (%)	17.3/22.3	RMS deviations (bonds/angles)	0.01 / 1.24
Ramachandran favored/outliers (%)	98/0	Poor rotamers (%)	12.4
Cβ deviations (>0.25 Å)	0.25%	Bad backbone bonds/angles (%)	0 / 0.25

^a^. Values within parentheses refer to the highest shell.

^b^. R_sym_ = ∑∑|Ihkl—Ihkl(j) |/∑Ihkl, where Ihkl(j) is observed intensity and Ihkl is the final average value of intensity.

^c^. Protein geometry analysis performed by MolProbity^56^.

^d^. R_work_ = ∑||Fobs| - |Fcalc||/∑|Fobs| and Rfree = ∑||Fobs| - |Fcalc||/∑|Fobs|, where all reflections belong to a test set of 10% data randomly selected in Phenix.

### Modelling *F*. *tularensis* SspA and SspA•MglA structures

A model of *F*. *tularensis* SspA was generated from its protein sequence using Phyre 2 [36]. Briefly, Phyre2 predicts a protein structure from its sequence using homology modelling. A profile for the protein is generated with PSI-blast, and then analyzed for secondary structure with PSI-pred. The success of the modelling is determined largely by how well the structure can be predicted with sequence alignments. Phyre2 is able to generate a reasonable model with a protein such as SspA, where several structures and homologous sequences are available. The resultant *F*. *tularensis* SspA model was then aligned in PyMOL onto the B subunit of the MglA dimer to generate the SspA•MglA heterodimer model.

### Protein Data Bank accession code

The MglA coordinates and structure factors have been deposited in the RCB Protein Data Bank under the accession code 4PUR. This work has been retrospectively approved by the Duke Institutional Biosafety Committee.

## Results and Discussion

### Overall MglA structure

**Fig 1:**
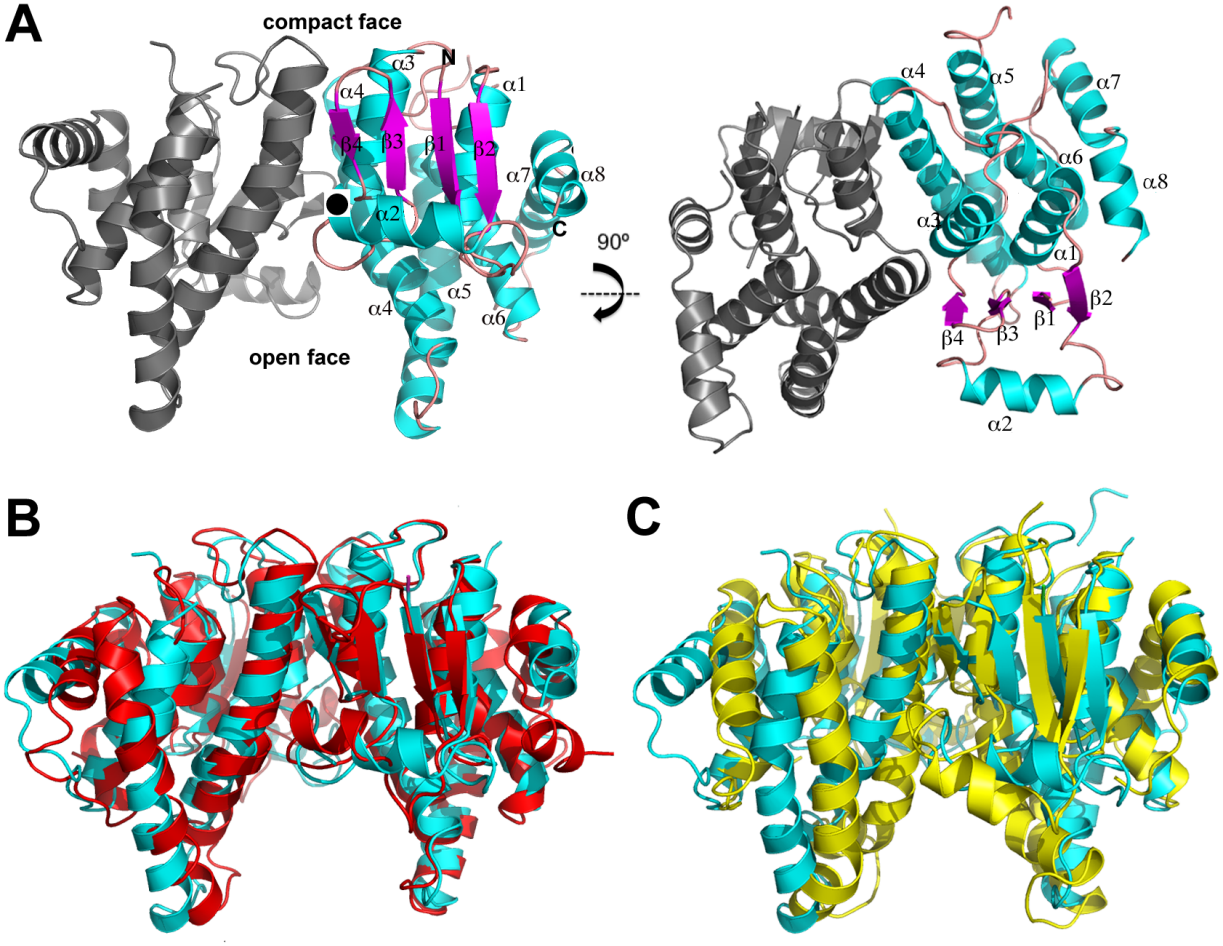
Structure of MglA and comparsion to SspA and GST proteins. A) Homodimeric MglA shown from two angles. The right subunit of the dimer is labelled and colored according to its β strands and α helices. B) Superimposition of the MglA dimer (cyan) onto the dimer of *Y*. *pestis* SspA (red). C) Superimposition of the MglA dimer (cyan) onto the dimer of nematode *C*. *elegans* specific GST (yellow). Although unexpected, MglA forms a dimer that is structurally similar in its arrangement to SspA dimers.

To gain insight into the function of the *F*. *tularensis* MglA protein we determined its structure by multiple wavelength anomalous diffraction (MAD). The crystal contains two molecules of MglA in the asymmetric unit (ASU), which form a dimer. The structure was refined to final R_work_ and R_free_ values of 17.3% and 22.8%, respectively to 2.95 Å resolution. Each MglA subunit harbors four β strands and 8 α helices (Fig 1A). The N-terminal region has a βαβαββα topology: β1 (residues 1–5)-α1 (residues 9–23)-β2 (residues 25–31)-α2 (residues 33–47)-β3 (residues 54–57)-β4 (residues 60–62)-α3 (residues 64–76). Following the N-terminal region is an all α helical, C-terminal domain with 5 helices: α4 (residues 85–111)-α5 (residues 116–142)-α6 (residues 156–172)-α7 (residues 180–191)-α8 (residues 193–201). MglA shows the strongest sequence homology to SspA proteins. However, the sequence identity between MglA and these proteins is only ~25%. Despite this low identity, Dali searches revealed that the MglA is structurally most similar to SspA proteins whereby comparison of the structure of the MglA subunit with those of *Y*. *pestis* SspA (PDB ID: 1YY7) [25], *P*. *putida* SspA PDB ID: 3MDK), and *P*. *fluorescens* SspA (PDB ID: 3LYP), resulted in root mean square deviations (rmsd) of 2.4 Å, 1.9 Å and 2.0 Å, respectively [37]. Thus, our structure unveils MglA as a new member of the SspA subclass of GST proteins.

Interestingly, MglA formed a dimer in the crystal. Formation of the MglA dimer buries an extensive surface area (BSA) of 2048 Å^2^, which is similar to the BSA observed in physiologically relevant oligomers, including GST dimers, which have BSAs ranging from ~2300 to 3200 Å^2^) [38]. MglA homodimerization is primarily electrostatic and is mediated by 14 salt bridges formed between residues on α4 and α5 (Fig 1A). Specifically, Arg64, Lys87, Arg89, and Arg93 interact with Asp59', Glu70', Asp74' and Glu97', respectively (where ' indicates the other subunit in the dimer) (S1 Fig). The mode of homodimerization of MglA is similar to that of SspA proteins as the dimerization interfaces of *Y*. *pestis*, *P*. *putida*, and *P*. *fluorescens* SspA proteins are also primarily polar in nature. For example, in *Y*. *pestis* SspA, Arg96, Arg100, Arg109, Arg105, Glu81 and Tyr56 interact with Glu81', Tyr56', Arg96', Arg100', Arg105' and Asp109', respectively (S1 Fig) [25]. Superimposition of the MglA dimer onto the homodimers of *Y*. *pestis* SspA, *P*. *putida* SspA and *P*. *fluorescens* SspA results in rmsds of 2.3 Å, 2.3 Å, and 2.5 Å, respectively (Fig 1B). By comparison, the MglA dimer is less analogous to canonical GST proteins; the MglA dimer superimposes with an rmsd of 4 Å onto the nematode *C*. *elegans* specific GST protein (Fig 1C). Indeed, similar to what is observed in SspA proteins, MglA dimerization is largely mediated by contacts between one region from each subunit. This leads to the creation of a V shaped morphology in the MglA dimer, whereby the tightly interacting regions of the dimer combine to create a compact surface (“compact face”) while the opposite side of the dimer harbors a large and exposed cavity (“open face”) (Fig 1A).

### Size exclusion chromatography and crosslinking studies

**Fig 2:**
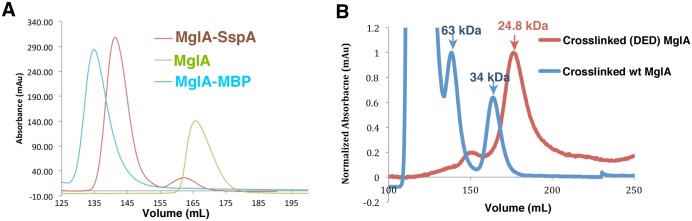
Size exclusion chromatography (SEC) and crosslinking analysis of MglA and SspA•MglA. A) S75 size exclusion chromatography profiles for MglA-MBP (blue), MglA (green) and MglA•SspA (red). MglA-MBP, MglA and SspA•MglA elute at 60 kDa (actual MW of monomer, 67 kDa), 27 kDa (calculated MW of a monomer, 24 kDa), and 51 kDa (calculated MW of the heterodimer, 48. kDa), respectively. B) SEC profiles from the S75 column for crosslinked MglA and the DED MglA mutant protein. Crosslinked wild type MglA (blue trace) elutes at 139 mL and 164 mL, corresponding to a MW of 63 kDa (dimeric MglA) and 34 kDa (monomeric MglA), respectively. Crosslinked DED MglA (red trace) elutes solely as a monomer. The y axis is the normalized absorbance (mAu) for the crosslinked proteins and the x axis is the elution volume.

Although the finding that MglA formed a dimer in the crystal was somewhat surprising, the BSA is similar to what is observed in functional oligomers, suggesting the possibility that MglA might form a physiologically relevant dimer. To assess if MglA can form homodimers in solution, we performed size exclusion chromatography (SEC). These analyses were performed on MglA at a concentration of 3 mg/mL and revealed that the protein was monomeric under our experimental conditions (Fig 2A). By sharp contrast, a mixture of SspA and MglA at a similar concentration eluted entirely as a dimer (Fig 2A).

The SEC experiments revealed that MglA is a monomer in solution under the conditions tested. However, if the MglA dimer is labile, it would not be revealed by these studies as oligomers that are weaker typically dissociate when subjected to separation-based techniques such as SEC. To test this hypothesis, MglA was crosslinked by glutaraldehyde, the reaction quenched and the resultant protein analyzed by SEC. Strikingly, the SEC profile showed two peaks, which corresponded to a monomeric (~34 kDa) and dimeric (~63 kDa) species of MglA (Fig 2B). The calculated molecular weight (MW) of monomeric MglA is ~24 kDa and dimeric MglA is 48 kDa and the small differences between the experimentally determined MW can be readily ascribed to the nonglobular shapes of the MglA monomer and dimer. Thus, the combined SEC and crosslinking data demonstrate that while MglA appears to preferentially heterodimerize with SspA, it can form weak homodimers in solution. This finding is consistent with previous two-hybrid studies, which demonstrated that MglA interacts with itself, but that the interaction was substantially reduced (7 to 8 fold) when compared to that observed in the same study between MglA and SspA [5]. However, two-hybrid analyses do not conclusively indicate a direct interaction nor do they demonstrate that the interaction involves specific dimer formation. Thus, to probe MglA dimerization further and specifically to assess whether the dimer observed in the crystal is that observed in our crosslinking studies, we generated mutations in the interface of the crystallographically observed MglA dimer. In particular, we mutated Asp59, Glu70 and Asp74, which the structure indicated are key to dimerization, to arginine residues and performed cross-linking and SEC on the mutant protein. The results revealed that MglA (D59R-E70R-D74R) protein did not form crosslinked dimers, supporting the notion that the crystallographic MglA dimer is also found in solution (Fig 2B).

### Putative small molecule binding site identified in MglA structure

**Fig 3:**
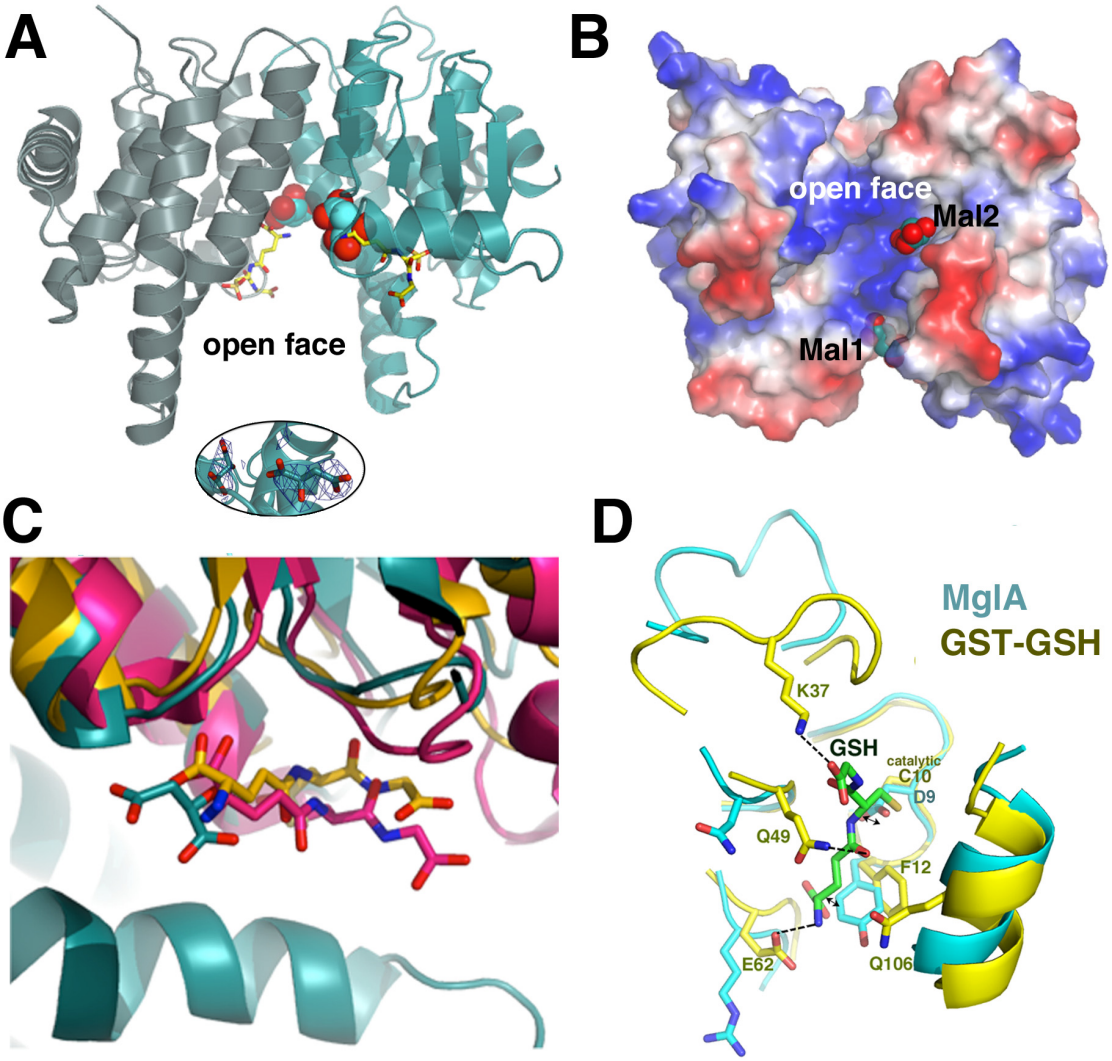
The MglA-malate binding pockets. A) Two malate molecules (shown as spheres) are bound in the large cleft of the open face of the MglA dimer. Also shown, as yellow sticks, are the corresponding binding sites of GSH molecules obtained by superimposition of the *E*. *coli* GST-GSH complex onto the MglA structure [42]. The inset below is a simulated annealing omit 2F_o_-F_c_ map (blue mesh), contoured at 1.0 σ and calculated after removal of the malate molecules followed by ten rounds of refinement using phenix.refine, with an initial temperature of 2500 K and a final temperature of 300 K. The map is contoured around the malate molecules. B) Electrostatic surface representation (where blue and red represent positive and negative surfaces, respectively) of the MglA dimer with bound malates (shown as sphere) included to underscore the electropositive nature of their binding region. C) Comparison of binding site of Mal1 (cyan sticks) in MglA with the GSH binding site of two GST proteins. For these analyses, the GST fold of MglA was aligned with those of the *E*. *coli* GST (yellow sticks) [42] and the *M*. *musculus* maleylacetoacetate isomerase (pink sticks) (PDB ID: 2CZ2). D) Superimposition of an MglA subunit onto that of the *N*. *gonorrhoeae* GST protein bound to GSH (PDB ID: 4HOJ). The subunits superimpose with an rmsd of 1.9 Å for 181 corresponding Cα atoms. The overlay shows that the catalytic cysteine of the GST protein is replaced by an aspartic acid in MglA, which clashes with the GSH as does the side chain of Tyr11. Moreover, a key GSH interacting loop and residues Lys37, Gln49, Glu62 and Gln106 are absent in the MglA structure.

The MglA structure was obtained using malate as a crystallization reagent and in the structure, electron density consistent with two malate molecules was found in the cleft within the open face of the dimer (Fig 3A [39]. The pocket in which the malates are bound is notably positively charged (Fig 3B). However, one malate, Mal2, is bound closer to positively charged residues, whereas the other malate, Mal1, is bound at the external face near the β sheet of the GST-fold (Fig 3B and 3C). The relatively low resolution of the structure makes the precise docking of the malate molecules into the density ambiguous and isothermal titration calorimetry experiments revealed no binding suggesting that the observed protein-malate interactions likely occur only at the high malate concentrations (2.1 M) used for crystallization.

Regardless, the finding that MglA interacts with malate, albeit only at higher concentrations, suggested the intriguing possibility that the open face of the dimer may function in binding small molecule ligands. Interestingly, in the Y. *pestis* SspA structure, citrate was used for crystallization and citrate molecules were bound similarly in this cleft [25]. Moreover, overlays of MglA onto GST-GSH structures show that the GSH molecules are also bound in the same cleft in which the malates bind (Fig 3A and 3C). However, analyses of these overlays indicate that MglA would likely not bind GSH nor would it catalyze the GST reaction as MglA is missing key residues that interact with GSH as well as those that are employed in the transferase activity by GST enzymes (Fig 3D). In particular, in place of the catalytic cysteine residue, MglA harbors an aspartic acid, which would clash with the GSH cysteinyl moiety (Fig 3D). Further, a key lysine that contacts the GSH is not present in MglA. Indeed, the region corresponding to the loop that harbors that lysine and encases the GSH adopts a different structure in MglA. However, to test whether MglA has any GST activity we carried out a dinitrobenzene conjugation-based assay. In these experiments, GST activity was measured for MglA, SspA•MglA and a GST control protein. The results revealed that MglA and SspA•MglA were not active as GSTs as they were unable to conjugate dinitrobenzene with reduced GSH, while the control GST protein displayed robust transferase activity (Table 2). We were unable to obtain enough purified, soluble *F*. *tularensis* SspA in the absence of MglA to assess its GST activity. However, it also lacks the GST catalytic residues strongly suggesting that this protein would not contain GST activity. Consistent with that hypothesis, as noted, previous studies have demonstrated that SspA proteins have no GST activity [25].

**Table 2:** GST activity measurement of *Francisella tularensis* MglA, SspA•MglA and pET41a GST.

Protein	GST activity ± SD
	*Activity units/mg protein*
GST	1.46 ± 0.01
MglA	0 ± 0.03
SspA•MglA	0.07 ± 0.09

### Mapping the RNA polymerase and PigR interacting surfaces on the SspA•MglA model and implications for small molecule ligand binding in regulator function

**Fig 4:**
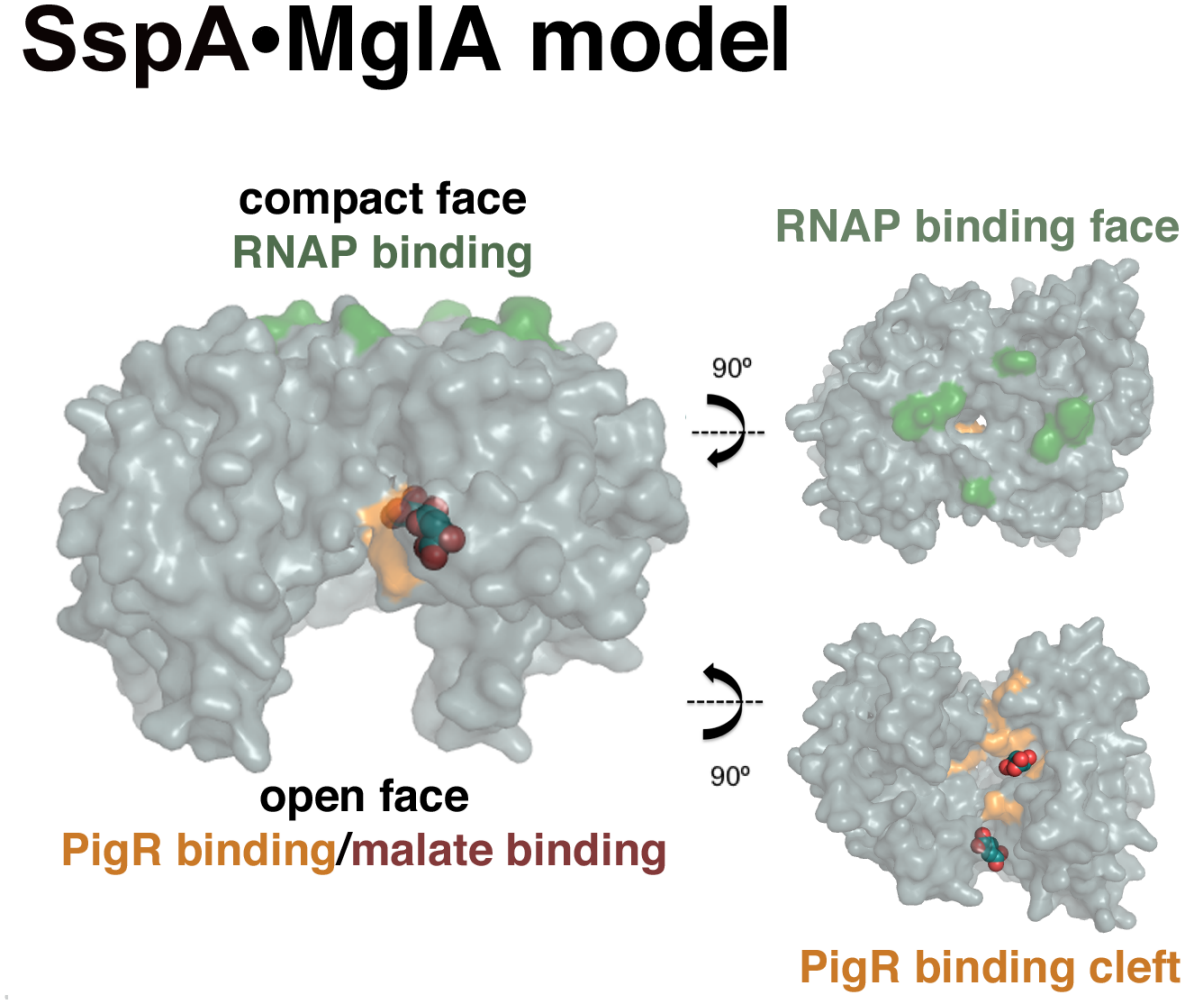
Model of the SspA•MglA heterodimer and visualization of its RNAP and PigR interacting surfaces relative to malate binding sites. (Left) The surface representation of the Phyre2 model of the SspA•MglA heterodimer with the residues involved in RNAP and PigR binding colored green and tan, respectively. Shown as spheres are the bound malates. Note, the malates bind in the same pocket as PigR. (Right) Views into the faces of the RNAP (top) and PigR/malate (bottom) binding pockets.

**Fig 5:**
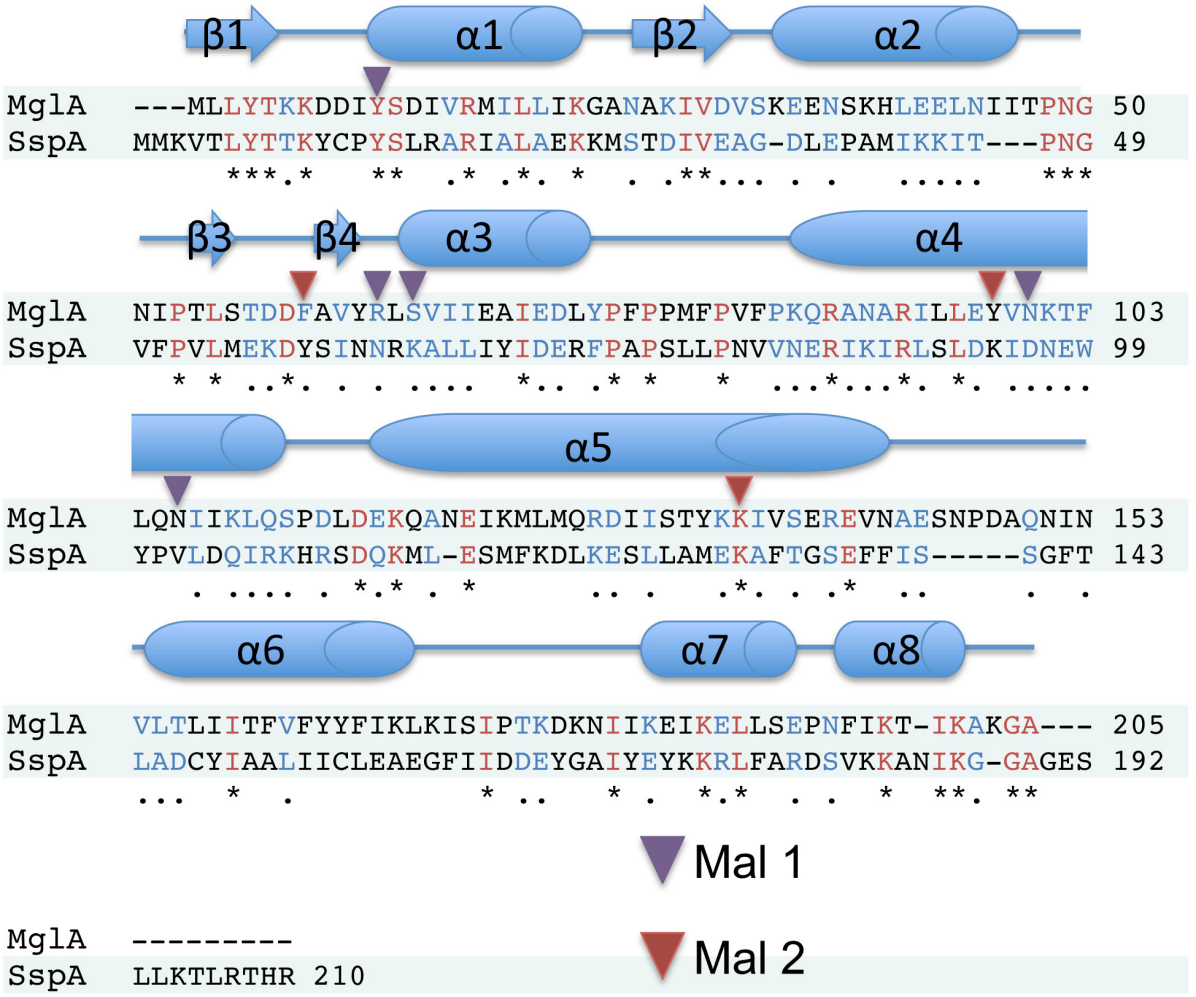
Sequence alignment of *F*. *tularensis* MglA and SspA. Shown above the sequence alignment is a cartoon depiction of the secondary structure of MglA. Residues that are identical between the proteins are shown in red and indicated with an asterisk, while similar residues are shown in blue and indicated with a period. Residues proximal to Mal1 and Mal2 are indicated by purple and red triangles, respectively.

Combined studies indicate that the SspA•MglA heterodimer binds both PigR, which is a putative DNA-binding MerR family member, and RNAP to coordinately control transcription of the FPI [5,6,13]. Further, two hybrid analyses indicate that the interacting regions of RNAP and PigR lie on opposite faces of the SspA•MglA heterodimer [13]. To gain insight into the binding sites of RNAP and PigR on the SspA•MglA heterodimer, we used Phyre 2, to generate an SspA•MglA heterodimer [36] (Fig 4). In *E*. *coli* SspA residues, Pro84, His85, Pro86, and Tyr92, were shown to be involved in RNAP binding. Several of these residues are conserved in MglA and SspA and map to the compact face of the SspA•MglA dimer (Fig 1A and 4). More recently, mutations in MglA and SspA that inhibit binding to PigR were determined [13]. Specifically, MglA residues Y11A, T47A, P48S, Y63A, Y64A, K101E all disrupted PigR binding and in SspA, a K65E substitution prevented PigR interaction. These mutations all map to the exposed, open face of the dimer (Fig 4). Notably, this region also encompasses the malate interaction sites in the MglA structure (Fig 4; Fig 5). This finding is particularly striking as recent studies have revealed that the anionic molecule, polyphosphate (PolyP), interacts directly with SspA•MglA and stabilizes the heterodimer [40]. In fact, PolyP activation of SspA•MglA may explain the previously observed stimulatory effects on FPI transcription caused by (p)ppGpp as studies demonstrated that the activity of the PolyP generating enzyme, PPX, is allosterically modulated by (p)ppGpp [40,41]. These combined data led Wrench *et al*. to propose that fluctuations in (p)ppGpp levels in *F*. *tularensis* affect the intracellular concentration of PolyP, which in turn affects the stability and activity (through PigR) of SspA•MglA. Our finding that malate, which like PolyP is an anionic molecule, binds in the same cavity of the SspA•MglA dimer as PigR, suggests the intriguing possibility that PolyP or other anionic ligand binding to this cleft may serve as a cofactor for PigR binding. Future studies will be needed to test this hypothesis.

## Conclusions

*F*. *tularensis* is one of the most virulent bacteria. Its virulence is determined by a unique pathogenicity island, which is under control of SspA and MglA. These proteins bind RNAP and the transcription regulators PigR and PmrA. While the *F*. *tularensis* SspA protein shows significant sequence homology to other SspA proteins, MglA is unique to *F*. *tularensis*. Thus, to gain insight into the structure and function of this critical *Francisella* virulence factor we solved its structure and performed a series of biochemical studies. These data reveal that MglA is structurally similar to SspA proteins. Crystal packing revealed an MglA dimer that is highly similar in its arrangement to SspA dimers, with compact and open faces. Biochemical data suggest that MglA exists as a weak dimer in solution but that it preferentially heterodimerizes with SspA. Finally, MglA crystallized with malate bound to a cationic pocket of the open face of the dimer, which also contains the determinants required for PigR interaction and overlaps the GSH binding pocket of GST. Although we determined that MglA and SspA•MglA do not function as GSTs, small molecule ligand binding by these proteins may play a role in modulating their interactions with target proteins.

## Supporting Information

S1 Fig Interaction networks at the dimerization interfaces of *F*. *tularensis* MglA, *Y*. *pestis* SspA, and *P*. *putida* SspA. The MglA interface results in a BSA of ~2000 Å^2^, *Y*. *pestis* SspA in ~2300 Å^2^, and *P*. *putida* SspA in ~2200 Å^2^. Salt bridges are shown by solid red lines, hydrogen bonds by solid blue lines, and non-bonded contacts as dashed yellow lines.
(DOC)
