# Supplementary material for: Structural and Biochemical Characterization of the Francisella tularensis Pathogenicity Regulator, Macrophage Locus Protein A (MglA)
Source: PLoS One. 2015 Jun 29;10(6):e0128225. doi: 10.1371/journal.pone.0128225 (PMC4488300; doi:10.1371/journal.pone.0128225)

**Fig. S1**. Interaction networks at the dimerization interfaces of *F. tularensis* MglA, *Y. pestis* SspA, and *P. putida* SspA. The MglA interface results in a BSA of ~2000 Å^2^, *Y. pestis* SspA in ~2300 Å^2^, and *P. putida* SspA in ~2200 Å^2^. Salt bridges are shown by solid red lines, hydrogen bonds by solid blue lines, and non-bonded contacts as dashed yellow lines.


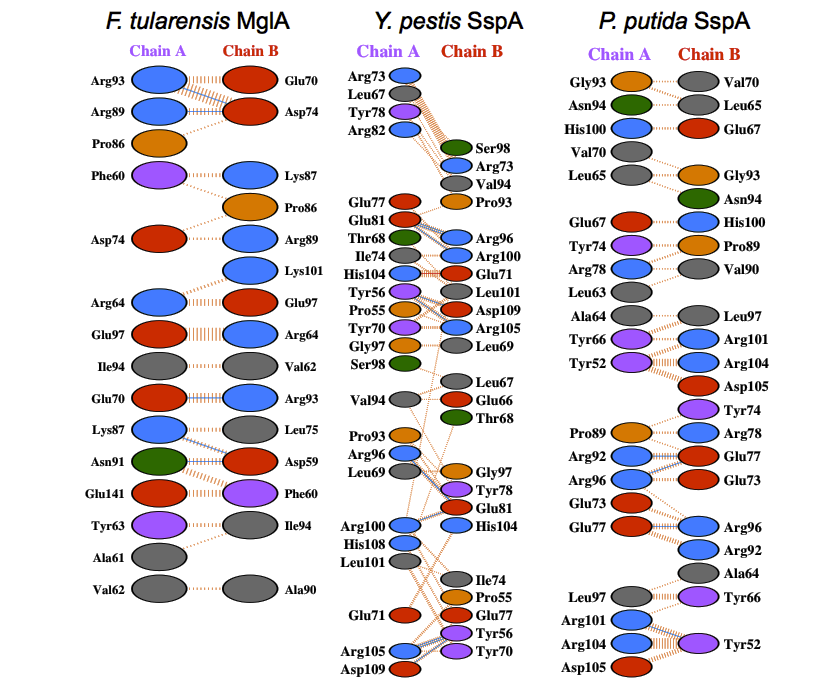

Supplement: S1 Fig — The MglA interface results in a BSA of ~2000 Å2, Y. pestis SspA in ~2300 Å2, and P. putida SspA in ~2200 Å2. Salt bridges are shown by solid red lines, hydrogen bonds by solid blue lines, and non-bonded contacts as dashed yellow lines. (DOC) [file pone.0128225.s001.doc]
